# Liposomal Encapsulated FSC231, a PICK1 Inhibitor, Prevents the Ischemia/Reperfusion-Induced Degradation of GluA2-Containing AMPA Receptors

**DOI:** 10.3390/pharmaceutics13050636

**Published:** 2021-04-30

**Authors:** Lindsay M. Achzet, Fanny Astruc-Diaz, Phillip H. Beske, Nicholas R. Natale, Travis T. Denton, Darrell A. Jackson

**Affiliations:** 1 Department of Pharmaceutical Sciences, Washington State University Health Sciences, Spokane, WA 99202, USA; Lindsay.achzet@wsu.edu (L.M.A.); travis.denton@wsu.edu (T.T.D.); 2 Department of Biomedical and Pharmaceutical Sciences, The University of Montana, Missoula, MT 59812, USA; fanny.diaz@mso.umt.edu (F.A.-D.); phillip.beske@umontana.edu (P.H.B.); Nicholas.natale@umontana.edu (N.R.N.); 3 Department of Biomedical Sciences, Elson S. Floyd, College of Medicine, Washington State University Health Sciences, Spokane, WA 99202, USA; 4 Steve Gleason Institute for Neuroscience, Washington State University Health Sciences, Spokane, WA 99202, USA

**Keywords:** ischemic/reperfusion injury, protein interacting with C kinase 1 (PICK1), AMPA receptor, FSC231, liposome, drug delivery system

## Abstract

Strokes remain one of the leading causes of disability within the United States. Despite an enormous amount of research effort within the scientific community, very few therapeutics are available for stroke patients. Cytotoxic accumulation of intracellular calcium is a well-studied phenomenon that occurs following ischemic stroke. This intracellular calcium overload results from excessive release of the excitatory neurotransmitter glutamate, a process known as excitotoxicity. Calcium-permeable AMPA receptors (AMPARs), lacking the GluA2 subunit, contribute to calcium cytotoxicity and subsequent neuronal death. The internalization and subsequent degradation of GluA2 AMPAR subunits following oxygen–glucose deprivation/reperfusion (OGD/R) is, at least in part, mediated by protein-interacting with C kinase-1 (PICK1). The purpose of the present study is to evaluate whether treatment with a PICK1 inhibitor, FSC231, prevents the OGD/R-induced degradation of the GluA2 AMPAR subunit. Utilizing an acute rodent hippocampal slice model system, we determined that pretreatment with FSC231 prevented the OGD/R-induced association of PICK1–GluA2. FSC231 treatment during OGD/R rescues total GluA2 AMPAR subunit protein levels. This suggests that the interaction between GluA2 and PICK1 serves as an important step in the ischemic/reperfusion-induced reduction in total GluA2 levels.

## 1. Introduction

Stroke and ischemic heart disease were responsible for 15.2 million deaths in 2016. Ischemic stroke is the second leading cause of death worldwide [1]. Ischemic stroke, the most prevalent form of stroke, occurs when blood flow is decreased or absent due to vessel occlusion [2]. While it is necessary to reintroduce blood flow to the infarcted area, this act also results in further damage by inflammation, oxidative stress, and delayed neuronal death (DND) within vulnerable neuronal populations, including CA1 hippocampal pyramidal neurons [3,4]. During ischemia, the lack of energy available disrupts ATP-dependent processes that maintain ionic gradients, which are critical to cellular function. Disrupting the ionic balance leads to excessive release of neurotransmitters, including glutamate, which are unable to be effectively cleared from the synapse [5]. Excessive stimulation of *N*-methyl-d-aspartate receptors (NMDARs) by glutamate is a contributing factor to DND [6,7,8,9], but multiple studies have reported that α-amino-3-hydroxy-5-methyl-4-isoxazolepropionic acid receptors (AMPARs) also contribute to DND [10,11,12,13,14,15].

AMPARs are ionotropic glutamate receptors in the brain and mediate fast excitatory neurotransmission. These receptors contain GluA2 subunits that render them impermeable to Ca^2+^ under physiological conditions, due to a post-transcriptional modification of glutamine residue 607, resulting in a charged arginine 607 residue that inhibits the passage of Ca^2+^ ions through the receptor [16]. As a result of ischemia/reperfusion, AMPARs undergo a subunit composition switch from Ca^2+^-impermeable, GluA2-containing AMPARs [15], to Ca^2+^-permeable, GluA2-lacking AMPARs. This allows the AMPAR to conduct calcium, which, in combination with excessive NMDAR stimulation, exacerbates cell death [17].

The internalization and subsequent degradation of the GluA2 subunit underlies this AMPAR subunit composition switch at the cell’s plasma membrane surface. The internalization of GluA2 subunits is, in part, mediated by protein interacting with C-kinase 1 (PICK1). PICK1 has a post-synaptic density protein (PSD)-95/Discs-large/zonula occludens (ZO)-1 (PDZ) binding domain which interacts with the C-terminal region of GluA2 and regulates its recycling in the cell [18]. Previous studies have observed that blocking the PICK1–GluA2 interaction leads to an increase in recycling of GluA2 back to the surface [19].

**Figure 1:**
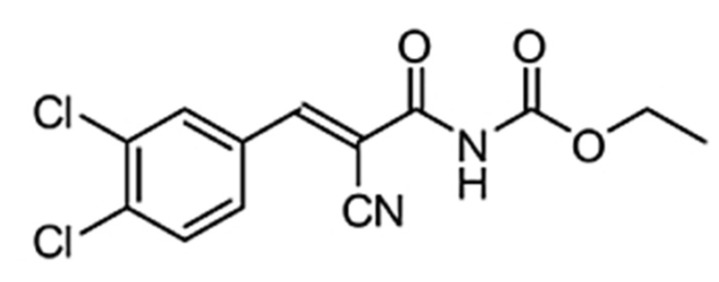
Structure of FSC231.

Recently, the first small-molecule inhibitor of PICK1, FSC231 (Figure 1), has been identified and shown to bind to the PDZ domain of PICK1 at an affinity similar to that observed for endogenous c-terminus of PICK1 peptides (K_i_ ~ 10.1 µM) [20]. However, the use of FSC231 as a PICK1 inhibitor has been limited due to its poor solubility in aqueous solutions.

Liposomes are widely recognized for being superior agents for drug delivery by solubilizing drugs and increasing their bioavailability [21]. Liposomes can also be modified for targeted delivery of drugs in a cell- or tissue-specific manner [22]. Furthermore, extensive toxicological screenings of liposomes have shown that they are remarkably safe for pharmaceutical use [23]. We sought to develop a drug-delivery system for FSC231 to use in our ex vivo animal model.

To evaluate the effect of FSC231 encapsulated in liposomes on the PICK1–GluA2 interaction, we utilized adult rat acute hippocampal slices exposed to oxygen–glucose deprivation and reperfusion (OGD/R), an in vitro model for ischemic/reperfusion injury. Here we demonstrate that FSC231 blocks the degradation of GluA2 by inhibiting the interaction of PICK1–GluA2.

## 2. Materials and Methods

### 2.1. Liposomal Formulation of FSC231

FSC231 was synthesized by using a previously published protocol [24]. In brief, a cyanoacetylcarbamic acid ethyl ester intermediate was synthesized from 2-cyanoacetic acid and urethane (ethyl carbamate), using the condensation agent phosphorous chloride oxide. The Knoevenagel reaction between cyanoacetylcarbamic acid and 3,4-dichlorobenzaldehyde was performed at room temperature, utilizing piperidine as a catalyst. Liposomes were prepared with FSC231, or without FSC231 (control), by solubilization in dimethyl sulfoxide (DMSO, 30 mg/g, *w*:*w*; FSC231/Lipid ratio 1:10)). Then 1,2-Dimyristoyl-sn-glycero-3-phosphocholine (DMPC; 50 mg/g in *tert*-butanol, *w*:*w*) and Tween-20 (1:10 tween-20/FSC231+DMPC, *w*:*w*) were added to the solution, and *tert*-butanol was added to make a total volume of 5 mL. The solution was frozen at −80 °C and then lyophilized over a period of 2 days. The thin film produced was a hygroscopic brittle glass. These physical properties indicate the complete removal of all solvents (water, *tert-*butanol, and DMSO). On the day of the experiment, the FSC231-lipid thin film was reconstituted in 5 mL of artificial cerebrospinal fluid (aCSF) or glucose-free aCSF by spinning the film in the presence of the solvent, in a 100 mL round-bottomed flask, connected to a rotary evaporator at atmospheric pressure (no vacuum), frozen with liquid nitrogen, reconnected to the rotary evaporator, and thawed to room temperature, with spinning, for ten cycles. The liposomal suspensions were diluted to a final concentration of 100 µM (based on mmol of FSC231) and kept at 4 °C until use in OGD/R experiments. Liposomes were characterized by liquid chromatography/mass spectrometry (LC/MS), dynamic light scattering (DLS), and transmission electron microscopy (TEM) and evaluated for encapsulation efficiency.

### 2.2. LC/MS

A total of 20 µL of FSC231 liposome suspension was added to 200 μL of ethanol. The instrument used was a HPLC/MS, Micromass LCT mass spectrophotometer composed of a Waters Alliance e2695 HPLC system that has a Waters 2998 photodiode array detector and orthogonal acceleration of TOF detection. A reverse-phase Phenomenex HPLC column (3µm NX-C18 110 Å, LC Column 50 × 4.6 mm, Waters, Milford, MA, USA) was used for the LC/MS analysis. Limit of detection (LOD) and limit of quantification (LOQ) were 0.1 and 0.3 mg/mL for DMPC, respectively. Baseline resolution of FSC231 was performed by a gradient elution at a flow of 0.6 mL/min with a gradient mobile phase composed of “mobile phase A” composed of water (0.8% trifluoroacetic acid) and “mobile phase B” composed of acetonitrile, ramped over a 15 min period, with an injection volume of 10 µL, monitored at 254 nm, using the following conditions: In the first three minutes, an isocratic run by 80% (*v*/*v*) mobile phase A; from 3.0 to 3.5 min, a linear gradient from 80% (*v*/*v*) mobile phase A to 55% (*v*/*v*) mobile phase A; from 3.5 to 5.0 min, a linear gradient from 55% (*v*/*v*) mobile phase A to 30% (*v*/*v*) mobile phase A; from 5.0 to 7.0 min, a linear gradient from 30% (*v*/*v*) mobile phase A to 0% (*v*/*v*) mobile phase A; from 7.0 to 12.0 min, an isocratic run at 0% (*v*/*v*) mobile phase A; from 12.0 to 15.0 min, a linear gradient from 0% (*v*/*v*) mobile phase A to 80% (*v*/*v*) mobile phase A.

### 2.3. Size Distribution and Surface Potential Measurements

Mean diameter, size distribution, polydispersibility index (PDI), and zeta potential of the liposomes were determined at 20 °C, by DLS (Zetasizer nano ZS, Malvern Instruments Ltd., Malvern, UK). The instrument was equipped with a He–Ne laser operating at a wavelength of 633 nm. Sizing measurements were made on the reconstituted liposomal samples diluted 1/100 (0.1 mg/mL) with 10 mM NaCl. Transmission electron microscopy (TEM) was performed, using an FEI Tecnai G2 20 Twin TEM (FEI, Hillsboro, OR, USA), to observe the average particle size and distribution of the samples. Then 2 µL of the liposomal suspension was placed on a formvar/carbon coated grid for 1 min, blotted off with a #4 filter paper wedge, and stained for 1 min with 2% uranyl acetate, followed by blotting with a #4 filter paper wedge. The grids were placed under a heat lamp for 5 min and then into a grid box and stored in a desiccator prior to imaging.

### 2.4. Encapsulation Efficiency

The encapsulation efficiency of the liposomes was determined by ultraviolet spectroscopy, using Eppendorf UVette cuvettes (VWR; Radnor, PA, USA) at a 1 cm pathlength, on an IMPLEN NanoPhotometer (Model NP80; Los Angeles, CA, USA). The liposomal suspension (200 µL) was mixed with 100% ethanol (200 µL), and the absorbance at 304 nm (λ_max_ FSC231, ε304 = 16,390 M^−1^·cm^−1^) was determined (Abs liposome solution (amount of free drug + amount of encapsulated drug)). Subsequently, 200 µL of the liposomal suspension was pelleted in an ultracentrifuge (100,000× *g*), at room temperature, for 60 min. The supernatant was discarded and the pellet was resuspended in 200 µL of aqueous NaCl (10 mM), and repelleted at 100,000× *g* for 60 min. The supernatant was discarded, the pellet was resuspended in aqueous NaCl (10 mM, 200 µL) and diluted with 100% ethanol (200 µL), and the absorbance at 304 nm was once again determined (Abs pellet solution (amount of encapsulated drug)). The encapsulation efficiency was determined by using the following equation:

$$\%\;\mathrm{encapsulation}\;\mathrm{efficiency}=\frac{\begin{array}{c} \mathrm{abs}\;\mathrm{pellet}\;\mathrm{solution}\\ \left(\mathrm{amount}\;\mathrm{of}\;\mathrm{encapsulated}\;\mathrm{drug}\right) \end{array}}{\begin{array}{c} \mathrm{abs}\;\mathrm{liposome}\;\mathrm{solution}\\ \left(\mathrm{amount}\;\mathrm{of}\;\mathrm{free}\;\mathrm{drug}+\mathrm{amount}\;\mathrm{of}\;\mathrm{encapsulated}\;\mathrm{drug}\right) \end{array}}\;\times 100$$

### 2.5. Animals

Adult (6–8 weeks old) male Sprague-Dawley rats (Charles River Labs, Wilmington, MA, USA) arrived at our facility on week 5. Rats were housed in a temperature-controlled facility with water and chow provided ad libitum. All animal protocols were approved by the University of Montana Institutional Animal Care and Use Committee.

### 2.6. Preparation of Acute Rat Hippocampal Slices

Adult male (6–8 week) Sprague-Dawley rats were anesthetized with isoflurane and rapidly decapitated. The brain was quickly removed and placed in an ice-cold cutting media (75 mM sucrose, 80 mM NaCl, 2.5 mM KCl, 1.25 mM NaH_2_PO_4_, 24 mM NaHCO_3_, 25 mM glucose, 4 mM MgCl_2_, 1 mM l-Ascorbic acid, 3 mM Na pyruvate, 0.5 mM CaCl_2_, pH 7.4). Both hippocampi were dissected, and coronal, 400-micron-thick slices were made by using a Mcllwain tissue chopper (Vibratome, St. Louis, MO, USA). Slices were equilibrated in oxygenated (95% O_2_, 5% CO_2_) aCSF (124 mM NaCl, 2.5 mM KCl, 1.25 mM KH_2_PO_4_, 26 mM NaHCO_3_, 10 mM glucose, 1.5 mM MgCl_2_, 2.5 mM CaCl_2_, pH 7.4), at 37 °C, for 60 min, prior to OGD/R.

### 2.7. OGD/R Procedure

Following equilibration, slices to be subjected to OGD/R were rinsed with glucose-free aCSF (aCSF with 10 mM mannitol substituted for 10 mM glucose, pH 7.4) and then transferred to deoxygenated glucose-free aCSF (0% O_2_, 95% N_2_, 5% CO_2_) and incubated for 40 min in a hypoxic glove box (Coy Laboratories, Grass Lake, MI, USA). Ten minutes prior to the end of OGD, FSC231-containing liposomes (100 µM, in deoxygenated, glucose-free aCSF) or DDS (drug delivery system, empty liposomes) were added to the slices. The slices were then transferred to oxygenated aCSF containing FSC231 (100 µM in aCSF), DDS, or no liposomal treatment for the time periods indicated. Normoxic controls were left in oxygenated aCSF for the duration of the experiments and were time-matched to the last reperfusion time-point of OGD-subjected slices.

### 2.8. Lysate Preparation

Slices were removed from aCSF at indicated reperfusion times and rinsed 3 times with ice-cold phosphate-buffered saline (PBS). Slices were transferred into tubes containing lysis buffer (250 mM sucrose, 20 mM HEPES, 2 mM EDTA, 5 mM MgCl_2_, 1 mM dithiothreitol, 1 mM 4-(aminoethyl)benzenesulfonyl fluoride hydrochloride (AEBSF), 1% protease and phosphatase inhibitor cocktail (Thermo, Rockland, IL, USA), 1% Triton X-100, 0.01% (*w*/*v*) saponin, pH 7.4) and lysed immediately by sonication 3 times, for 5 s, at 25% power, with a VirTis Ultrasonic Cell Disrupter 100 (Gardiner, NY, USA). Samples were centrifuged at 13,000× *g*, for 10 min, to remove cellular and nuclear debris. The supernatant was collected, and a bicinchoninic acid assay (BCA; Thermo, Rockland, IL, USA) was performed to determine protein concentration.

### 2.9. Western Blotting

Samples were denatured in Laemmli buffer/β-mercaptoethanol and heat (100 °C) for 10 min and resolved via sodium dodecyl sulfate polyacrylamide gel electrophoresis (SDS-PAGE). Samples were transferred to a nitrocellulose membrane (Bio-Rad, Berkeley, CA, USA) for subsequent detection, using immunoblotting. Blots were blocked for 1 h at room temperature with 5% (*w*/*v*) nonfat dry milk in tris buffered saline (0.1% *v*/*v* Tween-20, pH 7.5 (TBS-T)). Blots were then incubated with the following primary antibodies, overnight, at 4 °C: anti-PICK1 (1:1000, rabbit IgG, Epitomics, Burlingame, CA, USA); anti-GluA2 (1:1000, rabbit IgG, AbCam, Cambridge, MA, USA); anti GluA1 (1:1000, rabbit IgG, Epitomics, Burlingame, CA, USA); anti-β-Actin (1:5000, mouse IgG, Calbiochem, San Diego, CA, USA). Then they were washed 3 times with TBS-T and incubated with rabbit or mouse horseradish peroxidase secondary antibody (1:2000, Jackson ImmunoResearch, West Grove, PA, USA), for 2 h, at room temperature. Blots were then washed 3 times in TBS-T and then incubated with chemiluminescence substrate (Thermo, Rockland, IL, USA). Immunoreactive bands were visualized and captured with a Fuji Imaging System. Blots were stripped and re-probed up to 2 times, using Restore Plus Western Blotting Stripping Buffer (Thermo, Rockland, IL, USA).

### 2.10. Immunoprecipitation

Rat hippocampal slices were prepared as described above. Rat hippocampal slices were lysed in a buffer containing 50 mM Tris-HCl, 100 mM NaCl, 5 mM EDTA, 1 mM phenylmethylsulfonyl fluoride, 0.5% Triton X-100, and 1% protease and phosphatase inhibitor cocktail. Protein concentrations were determined by using a BCA assay, and lysates (500 µg/sample in 500 µL) were pre-cleared by using Protein-A/G 50/50 mix agarose beads (Bio-Rad, Redmond, WA, USA) for 1 h, at 4 °C, followed by incubation with a PICK1 antibody (15 µg per 500 µg protein, rabbit IgG, Epitomics, Burlingame, CA, USA), overnight, at 4 °C. The immunocomplex was then incubated for 4 h with 50 µL Protein-A/G 50/50 mix agarose beads, at 4 °C, with rotation, and washed 3 times with lysis buffer. Samples were eluted from the agarose beads by treatment with Laemmli buffer/β-mercaptoethanol and heat (100 °C) for 5 min and then subjected to SDS-PAGE. After transfer to nitrocellulose membranes, blots were blocked and incubated as described in Section 2.9, “Western Blotting”.

### 2.11. Statistical Analyses

The statistical analyses were performed by using GraphPad Prism 8 Software (Prism 8.4.0, 2020, GraphPad, San Diego, CA, USA). The *p*-values were calculated by using One-Way Analysis of Variance (ANOVA) with post hoc Sidak. Data are expressed as mean ± standard error of the mean (SEM). The results presented here are of at least three independent experiments.

### 2.12. Data Availability

All data presented in this paper are contained within the manuscript or available upon request of the corresponding author—D.A.J.

## 3. Results

### 3.1. Characterization of FSC231-Containing Liposomes

After initial rounds of experiments, FSC231 was found to possess poor solubility in aCSF solutions. To improve the solubility characteristics of FSC231, we sought to utilize a liposomal drug-delivery system.

Empty and FSC231-loaded liposomal suspensions were obtained by thin-film rehydration, using 10 mM aqueous NaCl (100 uM FSC231 final concentration), followed by 10x freeze-dry/thaw cycles to yield liposomal solutions with a liposomal average mean diameter of 173 nm (PDI 0.3) and 256 nm (PDI 0.3) (Table 1; Supplementary Figure S1), for the empty and FSC231-loaded liposomes, respectively. The average zeta potential was determined to be −2.89 and −7.01 for the empty liposomes and the FSC231-loaded liposomes, respectively (Table 1; Supplementary Figure S1). Utilizing UV–Vis and LC/MS, we examined the stability of FSC231-loaded liposomes over a 1-month period. The FSC231 was chemically stable over 1 month when stored as a lyophilized powder at 4 °C (Supplementary Figure S2).

**Figure 2:**
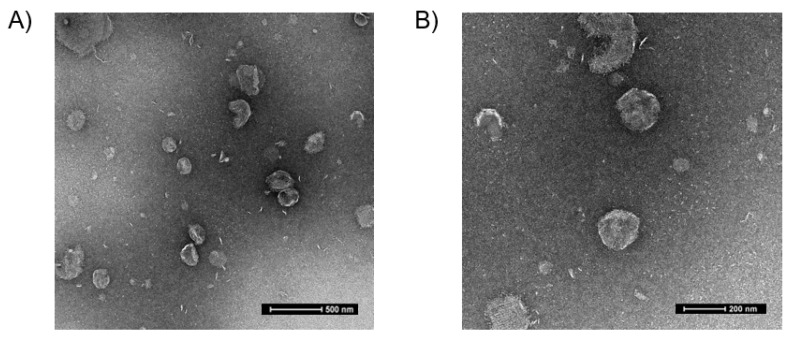
Representative transmission electron micrographs (TEM) of the liposomes. TEMs of the liposomes stained with uranyl acetate. (**A**) Representative TEM (lower magnification) demonstrating round shape and size with scale of 500 nm. (**B**) Representative TEM (higher magnification) demonstrating round shape and size with scale of 200 nm. The liposomes are roughly 250 nm in size, confirmed by dynamic light scattering.

The encapsulation efficiency of the FSC231-loaded liposomes was obtained by utilizing UV spectrophotometry (Supplementary Figure S3). The encapsulation efficiency was determined to be 58% (Table 2). Utilizing TEM, the FSC231-loaded liposomes were visualized to be fairly round in shape and size (~250 nm) (Figure 2), as confirmed by dynamic light scattering (Table 1).

A concentration of 100 µM FSC231 was chosen as a drug-saturating concentration which is approximately 10 times the K_i_ (10.1 µM) of FSC231 for the PDZ binding domain of PICK1. This concentration was subsequently used in all the following experiments. The bin/amphiphysin/rvs (BAR) domain on PICK1 has been suggested to bind to liposomes of specific size [25]. Though the exact radius of liposomes capable of binding with BAR have not been elucidated, we performed all subsequent experiments in the presence of empty liposomes to determine if there were any BAR associated effects, as shown in the following figures. We found no changes between untreated controls and our empty liposome drug-delivery system with regards to the PICK1–GluA2 interaction. This suggests that the empty liposomes alone have no effect on the PICK1–GluA2 interaction.

### 3.2. FSC231 Treatment Antagonizes NMDA-Induced PICK1–GluA2 Interaction

**Figure 3:**
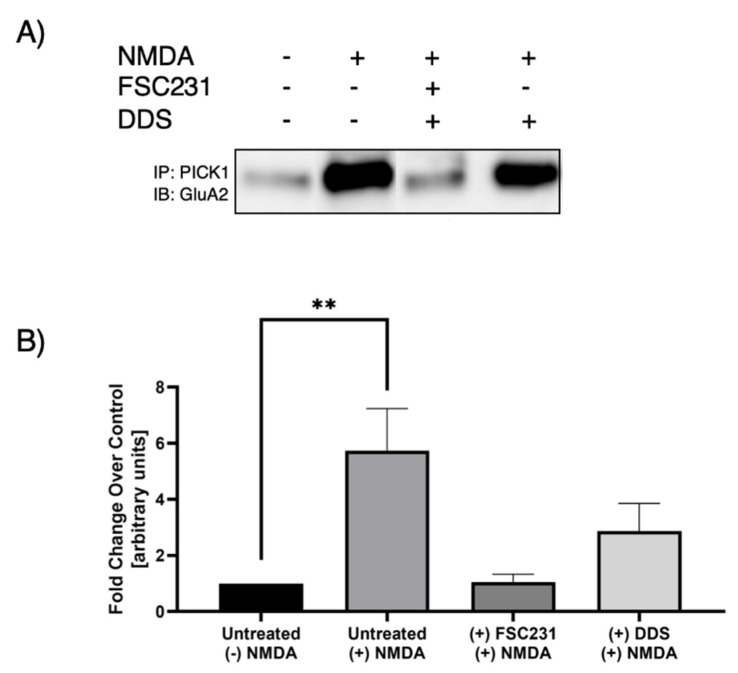
FSC231 prevents NMDA-induced association of GluA2 and PICK1. Fifteen minutes after hippocampal slices underwent 10 min NMDA (20 µM) stimulation, there was an increase in GluA2 association with PICK1. FSC231 treatment prevents this increase in association, whereas empty liposome drug-delivery system (DDS) does not, in hippocampal slices. (**A**) Immunoprecipitation of PICK1, and representative Western blot for GluA2. (**B**) Quantification of Western blots normalized to the unstimulated, untreated samples. N = 3, one-way analysis of variance (ANOVA) with post hoc Sidak; ** denotes *p* < 0.01; data are expressed as mean ± standard error of the mean (SEM).

Previous studies have observed that NMDAR activation leads to increased interaction between PICK1 and GluA2 [18]. Treatment with FSC231 blocked the NMDA-induced increase in association between PICK1 and GluA2 (Figure 3). As a positive control, we have shown that FSC231 is able to inhibit NMDA-mediated PICK1–GluA2 association (Figure 3).

### 3.3. Optimal PICK1–GluA2 Association

**Figure 4:**
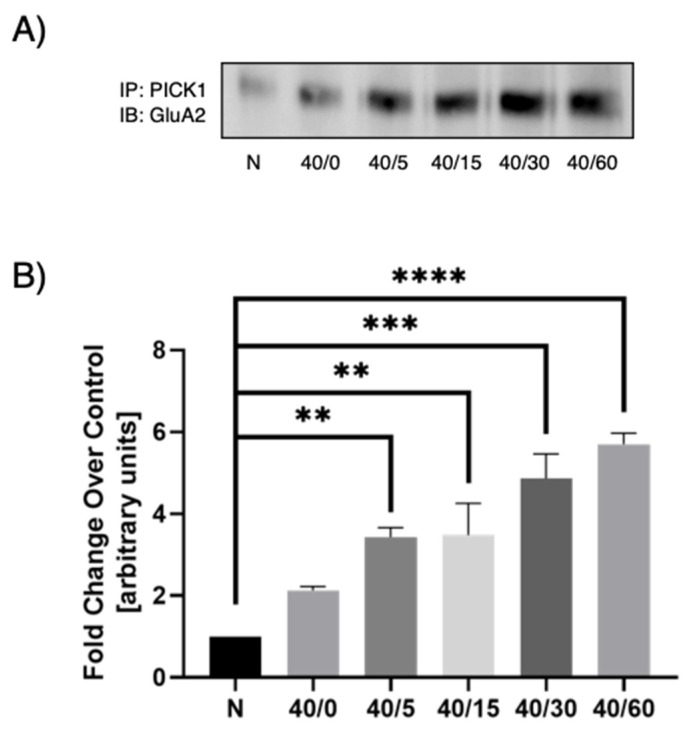
OGD/R-induced PICK1–GluA2 interaction increases with reperfusion time. Post-OGD/R immunoprecipitation of PICK1 and subsequent immunoblotting for GluA2 show an increase in PICK1–GluA2 interaction in hippocampal slices at 40 min of OGD and 5, 15, 30, and 60 min of reperfusion. (**A**) Representative Western blot of GluA2 following immunoprecipitation of PICK1 at indicated OGD/R time-points. (**B**) Quantification of Western blots normalized to normoxic control. N = 3, one-way ANOVA with post hoc Sidak, ** denotes *p* < 0.01, *** denotes *p* < 0.001, **** denotes *p* < 0.0001, data are expressed as mean ± standard error of the mean (SEM).

Previous studies under OGD conditions suggests that the GluA2 AMPAR subunit composition switch is dependent on PICK1 trafficking [26] and that this trafficking contributes to delayed neuronal death in OGD-induced hippocampal slices [19]. These conditions have not been examined under reperfusion conditions. To determine the time point at which to carry out our OGD/R experiments, we first conducted a time-response experiment in which we used 40 min OGD and increasing reperfusion times (0, 5, 15, 30, or 60 min). In previous studies [27], 40 min of OGD was determined to be optimal in hippocampal slices. PICK1 was immunoprecipitated from all samples and then immunoblotted for GluA2. We found an increase in PICK1–GluA2 association with reperfusion when compared to the normoxic control, with maximal association between 30 and 60 min of reperfusion (Figure 4). A reperfusion time of 30 and 60 min gave statistically significant results. In subsequent experiments, the 30-min reperfusion time point was used.

### 3.4. FSC231-Containing Liposomes Attenuate OGD/R-Induced PICK1–GluA2 Interaction

**Figure 5:**
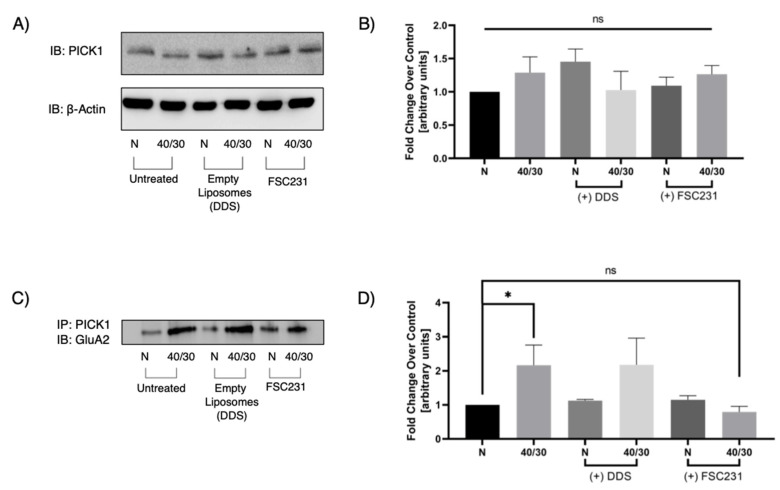
FSC231 prevents OGD/R-induced interaction of PICK1 and GluA2. (**A**) Representative Western blot of PICK1 indicating that PICK1 protein levels are unchanged with OGD/R in hippocampal slices. (**B**) Quantification of Western blots represented in (**A**). (**C**) Representative Western blot demonstrating that 40 min of OGD and 30 min of reperfusion increases the association between PICK1 and GluA2. FSC231 treatment prevents the OGD/R-induced increase in PICK1–GluA2 interaction. (**D**) Quantification of post-OGD/R immunoprecipitation of PICK1 and subsequent immunoblotting for GluA2 normalized the normoxic untreated control. N = 12, one-way ANOVA with post hoc Sidak; * denotes *p* < 0.05; ns denotes no significance.

No previous studies have evaluated PICK1 expression under OGD/R conditions. Increased PICK–GluA2 association could be attributed to an increased expression of PICK1. We therefore performed Western blot analysis on total lysate treated with or without FSC231 to investigate if total PICK1 expression was altered during 40/30 OGD/R. There was no significant change in PICK1 protein levels as a result of OGD/R treatment (Figure 5A). To investigate the ability of FSC231 to inhibit the PICK1–GluA2 interaction, we next performed immunoprecipitation experiments on OGD/R exposed hippocampal slices in the presence or absence of FSC231 and empty liposomes. We found that FSC231 treatment significantly reduced the PICK1–GluA2 interaction (Figure 5C). Furthermore, slices that were treated with empty liposomes exhibited no significant change in PICK1–GluA2 association compared to untreated controls. These results suggest that FSC231 may be blocking the PDZ binding domain of PICK1, preventing the interaction with GluA2.

### 3.5. FSC231 Treatment Prevents OGD/R-Induced Degradation of GluA2 AMPAR Subunit

**Figure 6:**
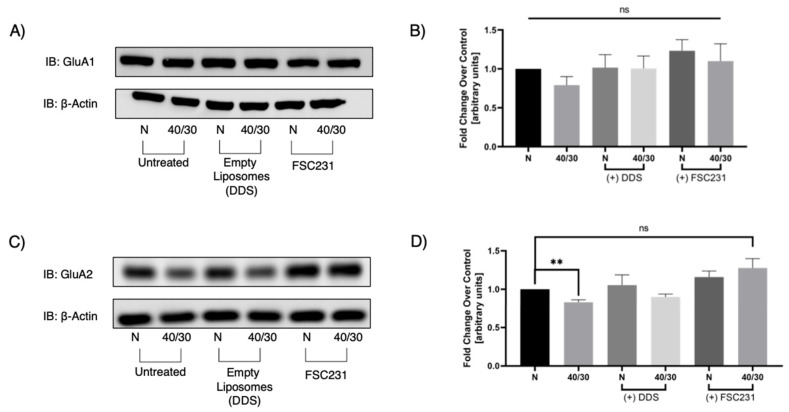
FSC231 treatment prevents the OGD/R-induced degradation of GluA2. (**A**) Representative Western blot for GluA1 indicating that 40 min of OGD and 30 min of reperfusion does not alter GluA1 protein levels in hippocampal slices. (**B**) Quantification of Western blots from (**A**). (**C**) Representative Western blot for GluA2 demonstrating that 40 min of OGD and 30 min of reperfusion decreases the amount of GluA2 protein in hippocampal slices. This effect is ameliorated with treatment of FSC231. (**D**) Quantification of immunoprecipitation/ immunoblotting experiments from (**C**). N = 12, one-way ANOVA with post hoc Sidak; ** denotes *p* < 0.01, ns denotes no significance, and data are expressed as mean ± standard error of the mean (SEM).

Since GluA2 AMPAR subunit trafficking is, at least in part, dependent on PICK1, we next sought to examine whether PICK1 is involved in the OGD/R-induced degradation of GluA2. Total GluA1 protein levels were unaffected by OGD/R, and FSC231 treatment also had no effect in GluA1 protein levels (Figure 6A). However, OGD/R-induced degradation of GluA2 was prevented in slices treated with FSC231 (Figure 6C).

## 4. Discussion

FSC231 has been utilized in studies examining pulmonary hypertension [28,29], acute liver injury [30], hyperalgesia [31,32], willed-movement in focal cerebral ischemia [33], and cocaine-seeking behavior [34]. This study is the first to analyze the therapeutic potential of this small molecule in ischemia/reperfusion injury. FSC231 binds to the PDZ domain of PICK1, hindering its ability to associate with the GluA2 AMPAR subunit.

Delayed neuronal death following OGD/R has been attributed to decreased surface expression of GluA2-containing AMPARs and increased expression of surface GluA2-lacking Ca^2+^-permeable AMPARs [10,11,12,13,14,15]. The present findings demonstrate that OGD/R-induced AMPAR trafficking is mediated by the PICK1 interaction with GluA2. Previous studies have shown that disrupting the PICK1–GluA2 interaction with a c-terminal GluA2 peptide (EVKI) effectively attenuates OGD-induced GluA2 internalization [19]. The present work aimed to observe this interaction with the use of the recently described PICK1 PDZ inhibitor, FSC231 [20], with the added implementation of reperfusion following OGD. While c-terminal peptide inhibitors possess higher selectivity due to mimicking natural binding partner sequences, they possess poorer pharmacokinetic profiles when compared to small molecules [35]. Compared to endogenous peptides, FSC231 was found to have a similar binding affinity to PICK1.

Consistent with previous findings using c-terminal peptide inhibitors of the PICK1 PDZ domain, we found a significant decrease in PICK1–GluA2 interaction with FSC231. It is unclear whether FSC231 completely blocks the internalization of GluA2-containing AMPARs or inhibits the slow recycling of internalized GluA2 back to the surface. Future studies are needed to evaluate the balance between endocytosis and exocytosis of AMPARs with OGD/R, and the effect of FSC231 on this tightly regulated process. PICK1 expressed in HEK293 cells was found to regulate the trafficking and recycling rate of its PDZ binding partners through a Rab11-dependent recycling pathway [36]. PICK1 knockout (KO) mice were shown to have no considerable difference in NMDA-induced internalization of GluA2 compared to wild-type neurons, but exhibited increased acceleration rates of GluA2 recycling to the neuronal plasma membrane [18].

The phosphorylation of GluA2 at the serine 880 residue has been shown to be important for the surface removal of GluA2. Serine 880 phosphorylation of GluA2 diminishes its affinity for glutamate receptor-interacting protein 1 (GRIP1), an AMPA binding protein (ABP) scaffold protein that localizes AMPARs to the synaptic membrane [37]. When GluA2 is phosphorylated at the serine 880 residue, it increases its affinity for binding to the PICK1 PDZ binding domain [37,38]. There are reports implicating PKCα as the kinase responsible for GluA2 phosphorylation at the serine 880 residue [39,40]. This phosphorylation is suggested to be facilitated by PICK1, acting as a scaffold, bringing PKCα to GluA2 by direct binding of PICK1 to ABP [39], but it is unclear if this mechanism holds true under OGD/R conditions. Recently, we provided evidence that the increase in serine 880 phosphorylation resulted in the dissociation of GluA2 from GRIP1/ABP, thus, enabling the association of GluA2 with PICK1 [41]. This study also reported that there is an increase in the association of activated PKCα with PICK1 following OGD/R [41].

**Figure 7:**
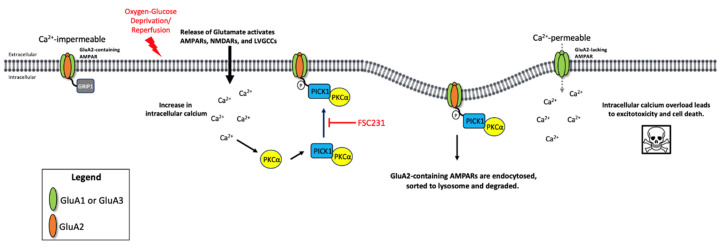
Potential mechanism of FSC231 treatment following OGD/R. Under normal physiologic conditions, AMPARs in the hippocampus are GluA2-containing Ca^2+^-impermeable receptors. GRIP1 is a scaffold protein that aids in tethering GluA2-containing AMPARs to the cell membrane. Under pathologic OGD/R conditions, there is a massive release of neurotransmitters, including glutamate, which activates AMPARs and subsequently activates NMDARs and l-type voltage-gated calcium channels. An increase in intracellular Ca^2+^ activates PKCα binding to PICK1, which translocates to the plasma membrane. PICK1 binds to GluA2, GluA2 is phosphorylated by PKCα, and is subsequently endocytosed and degraded. There is a subsequent increase in GluA2-lacking Ca^2+^-permeable AMPARs. This leads to intracellular calcium overload, resulting in excitotoxicity and cell death. FSC231 blocks the interaction of GluA2–PICK1, prevents the GluA2 AMPAR subunit from being degraded, and acts in a neuroprotective manner.

Further studies using specific pharmacological inhibitors and/or knockdown of PKCα are necessary to determine the exact role of PKCα in OGD/R. It is possible that inhibiting the interaction between PICK1 and PKCα with FSC231 may decrease phosphorylation of GluA2, resulting in less internalization, and subsequent degradation, of GluA2-containing AMPARs following OGD/R (Figure 7). However, this is speculative and further investigation is required.

Treatment with FSC231 antagonized the OGD/R-induced degradation of the GluA2 AMPAR subunit. It is unclear whether this is due to decreased endocytosis of the GluA2 AMPAR subunits following OGD/R or if this is a result of increased endosomal sorting to the slow recycling endosome. Either of these actions would result in GluA2 not being targeted towards a degradative pathway with OGD/R. Further studies are needed to confirm PICK1’s role in the endocytic trafficking and degradation of the GluA2 subunit with OGD/R.

PICK1 has been recently suggested to be a valuable drug target for the treatment of neurological disorders, including excitotoxicity [42], cancer [43], and neuropathic pain [44], through its PDZ binding domain. PICK1 has also been an ongoing subject of interest in research involving synaptic plasticity [45,46]. It will be important to understand the multiple roles this protein plays in the trafficking of its binding partners. FSC231 has the potential to be a small-molecule therapeutic for the prevention of ischemia/reperfusion-induced delayed neuronal death in vivo, by preventing the degradation of GluA2-containing AMPARs. Many studies have utilized a liposomal drug-delivery system to target the brain following ischemia/reperfusion in vivo; see the recent review from Fukuta et al. [47]. It has been previously demonstrated that PEGylated liposomes under 200 nm successfully targeted the brain in vivo and delivered Fasudil, a Rho-kinase inhibitor, to the ischemic region [48]. With these parameters in mind, it is possible that FSC231 may also translate to an in vivo therapy for ischemia/reperfusion injury, utilizing a liposomal system to deliver this therapeutic small molecule to the brain.

## Figures and Tables

**Table 1:** Summary of size distribution results. Empty MLVs had an average particle size of 173 nm, polydispersibility index (PDI) of 0.369 and zeta potential of −2.89 mV. MLVs encapsulating FSC231 were found to have an average particle size of 256 nm, PDI of 0.364, and zeta potential of −7.01 mV.

Sample	Article Particle Size (nm)	Polydispersibility Index (PDI)	Zeta Potential (mV)
Empty Multilamellar Vesicles	173.3	0.369	−2.89
FSC231-loaded Multilamellar Vesicles	256.1	0.312	−7.01

**Table 2:** Determination of encapsulation efficiency (%). The encapsulation efficiency of the liposomes was determined with UV–Vis by measuring the absorbance at 304 nm (bolded in table) and utilizing the equation $\frac{\mathrm{amount}\;\mathrm{of}\;\mathrm{encapsulated}\;\mathrm{drug}}{\mathrm{amount}\;\mathrm{of}\;\mathrm{free}\;\mathrm{drug}+\mathrm{amount}\;\mathrm{of}\;\mathrm{encapsulated}\;\mathrm{drug}}\times 100.$

Wavelength (nm)	Encapsulation Efficiency (%)
302	58
303	58
**304**	**58**
305	58
306	57

## Data Availability

All data are contained in the manuscript or available upon request to the corresponding author: D.A.J.; Darrell.jackson@wsu.edu.

## Supplementary Materials

The following are available online at https://www.mdpi.com/article/10.3390/pharmaceutics13050636/s1, Figure S1: Size distribution and zeta potential of FSC231-loaded MLVs, Figure S2: FSC231 is stable when encapsulated in MLVs as a lyophilized powder for up to at least 30 days, Figure S3: Representative UV scans for determination of encapsulation efficiency.
