# Supplementary material for: Liposomal Encapsulated FSC231, a PICK1 Inhibitor, Prevents the Ischemia/Reperfusion-Induced Degradation of GluA2-Containing AMPA Receptors
Source: Pharmaceutics. 2021 Apr 30;13(5):636. doi: 10.3390/pharmaceutics13050636 (PMC8146086; doi:10.3390/pharmaceutics13050636)
Supplement: Supplementary file 1 [file pharmaceutics-13-00636-s001.zip › pharmaceutics-1176607-supplementary.pdf]

pharmaceutics

MDPI

Supplementary Materials: Liposomal Encapsulated FSC231, a PICK1 Inhibitor, Prevents the Ischemia/Reperfusion-Induced Degradation of GluA2-Containing AMPA Receptors

Lindsay M. Achzet, Fanny Astruc-Diaz, Phillip H. Beske, Nicholas R. Natale, Travis T. Denton and Darrell A. Jackson

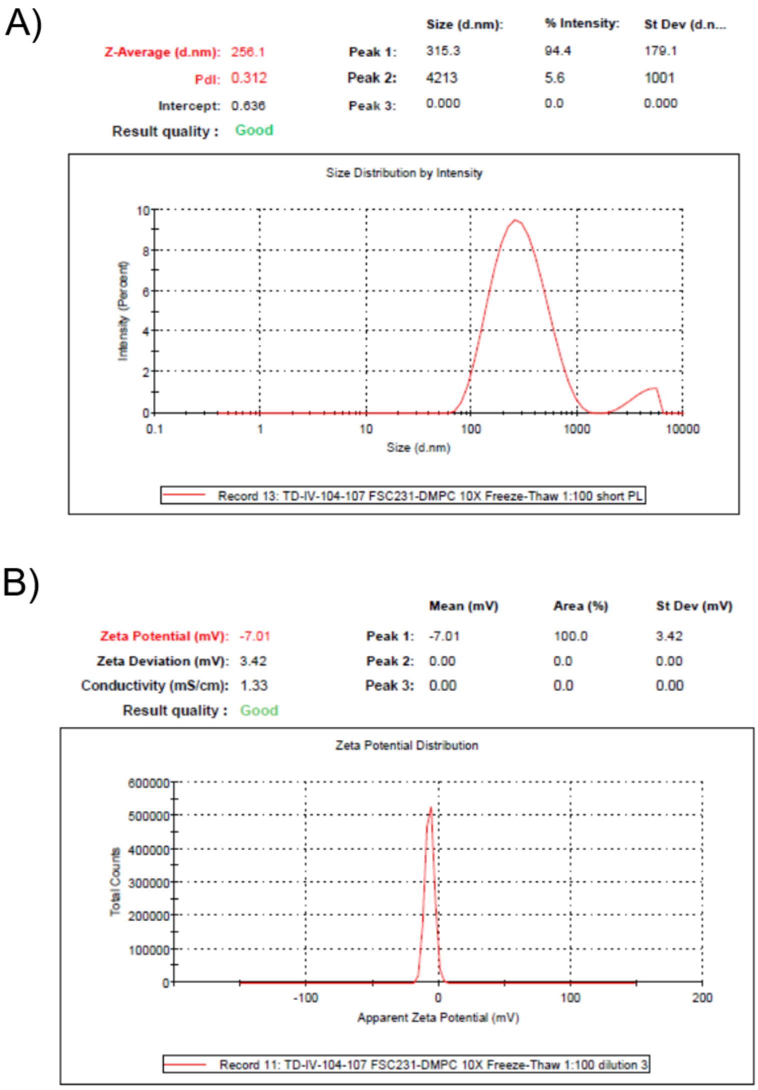

Figure S1. Size distribution and zeta potential distribution of FSC231-loaded MLVs. (**A**) The homogenized FSC231-loaded liposomes form a monodisperse suspension with an average of 256 nm and a reasonable PDI of 0.312. (**B**) The average zeta potential of FSC231-loaded MLVs is −7.01mV.

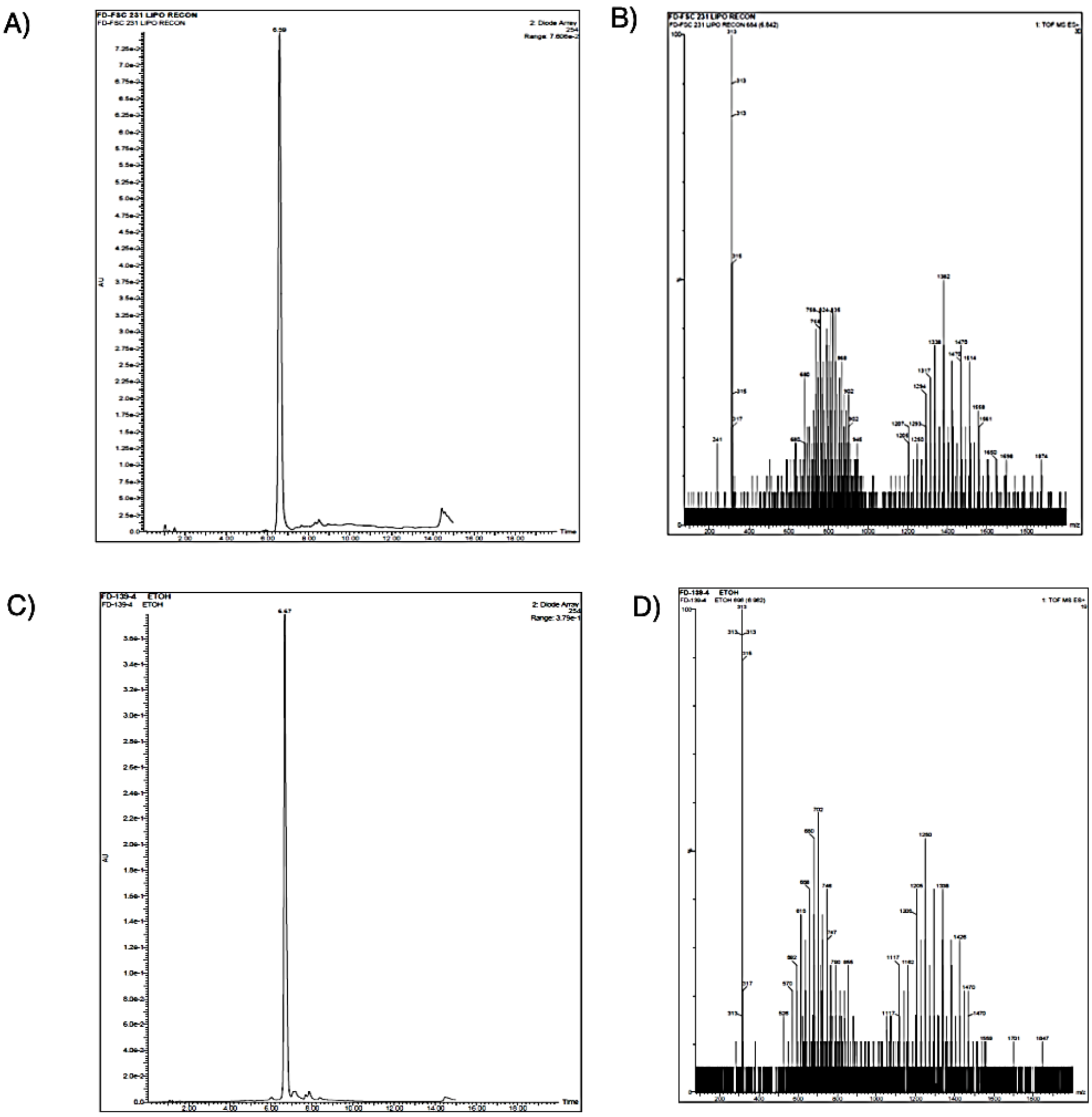

Figure S2. FSC231 is stable when encapsulated in MLVs as a lyophilized powder for up to at least 30 days. (**A**) UV-Vis spectrum. Freshly manufactured FSC231-loaded MLVs retention time was 6.67 minutes. (**B**) Liquid chromatography/mass spectrometry (LC/MS). Freshly manufactured FSC231-loaded MLVs m/z +1 was 313. (**C**) UV-Vis spectrum. FSC231-loaded MLVs stored as a lyophilized powder for 30 days at 4 °C retention time was 6.69 minutes. (**D**) LC/MS. FSC231-loaded MLVs stored as a lyophilized powder for 30 days at 4 °C m/z +1 was 313.

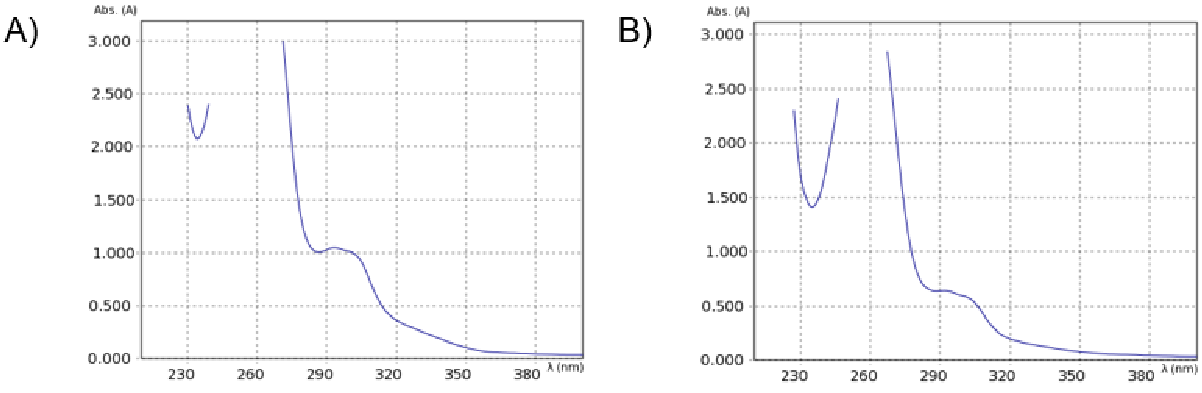

Figure S3. Representative UV scans for determination of encapsulation efficiency. (**A**) UV scan of the non-pelleted liposomal solution (1:1 10mM NaCl/EtOH). (**B**) UV scan of pelleted liposomal solution (1:1 10mM NaCl/EtOH).
